# Building a Compact Convolutional Neural Network for Embedded Intelligent Sensor Systems Using Group Sparsity and Knowledge Distillation

**DOI:** 10.3390/s19194307

**Published:** 2019-10-04

**Authors:** Jungchan Cho, Minsik Lee

**Affiliations:** 1 Department of Software, Gachon University, Seongnam 13120, Korea; thinkai@gachon.ac.kr; 2 Division of Electrical Engineering, Hanyang University, Ansan 15588, Korea

**Keywords:** convolutional neutral network, deep learning, group sparsity, knowledge distillation, parameter reduction

## Abstract

As artificial intelligence (AI)- or deep-learning-based technologies become more popular, the main research interest in the field is not only on their accuracy, but also their efficiency, e.g., the ability to give immediate results on the users’ inputs. To achieve this, there have been many attempts to embed deep learning technology on intelligent sensors. However, there are still many obstacles in embedding a deep network in sensors with limited resources. Most importantly, there is an apparent trade-off between the complexity of a network and its processing time, and finding a structure with a better trade-off curve is vital for successful applications in intelligent sensors. In this paper, we propose two strategies for designing a compact deep network that maintains the required level of performance even after minimizing the computations. The first strategy is to automatically determine the number of parameters of a network by utilizing group sparsity and knowledge distillation (KD) in the training process. By doing so, KD can compensate for the possible losses in accuracy caused by enforcing sparsity. Nevertheless, a problem in applying the first strategy is the unclarity in determining the balance between the accuracy improvement due to KD and the parameter reduction by sparse regularization. To handle this balancing problem, we propose a second strategy: a feedback control mechanism based on the proportional control theory. The feedback control logic determines the amount of emphasis to be put on network sparsity during training and is controlled based on the comparative accuracy losses of the teacher and student models in the training. A surprising fact here is that this control scheme not only determines an appropriate trade-off point, but also improves the trade-off curve itself. The results of experiments on CIFAR-10, CIFAR-100, and ImageNet32 × 32 datasets show that the proposed method is effective in building a compact network while preventing performance degradation due to sparsity regularization much better than other baselines.

## 1. Introduction

Embedded intelligence is characterized by the ability to deliver smart systems or services to the industry through the integration of software and hardware. This kind of embedded intelligence can serve many purposes, such as the smart monitoring of user-health or human behaviors and in systems to be used by the aged or the disabled. However, for the information collected from sensors to be used for such embedded intelligence, it is necessary for the sensor signal to be processed by machine learning [1,2,3,4,5]. The use of deep learning is the best choice for this task because deep networks have achieved state-of-the-art performance in many major machine learning areas, such as computer vision and natural language processing [6,7,8]. Moreover, deep learning has expanded beyond academic research into various commercial fields, which has been considered a quasi-revolution.

However, there are still problems to be solved for embedding deep-network-based functionality on intelligent sensors. A major problem is that the expressive power comes from the increased number of learnable parameters, making it impractical to use deep networks on limited platforms, such as mobile phones and robots, and on sensors with low-capacity processing power in devices like autonomous vehicles. Furthermore, a large number of parameters could lead to model overfitting, if there are insufficient training data [9].

Unfortunately, even though it is well known that deep neural networks have many redundant parameters [10,11] and can be replaced by more compact architectures, designing compact deep architectures for new tasks still remains a dark art: it requires the determination of the number of parameters or the complexity of the model, which can be a difficult problem itself. The complexity is typically set manually through trial and error, resulting in a hard trade-off between accuracy and the number of trainable parameters.

Several attempts have been made to overcome such difficulties in creating lightweight networks. One method is to transfer knowledge from a cumbersome (teacher) model to a smaller one (student network), which is more appropriate for limited platforms [12,13,14,15,16,17]. However, this method has a problem: it is assumed that the correct number of parameters for a student network is already known during the training phase.

Another approach to achieve a more compact deep network is to learn a compact structure by removing unnecessary weights from a large initial network (parameter reduction) [18,19,20,21]. For example, utilizing sparse regularizes, such as the $l_{1}$-norm [22] or $l_{2,1}$-norm [23], helps in reducing the redundant neurons or layers. However, a major problem with this approach is that it often achieves this efficiency at the expense of accuracy. How can we then obtain a compact network without sacrificing accuracy? It still remains an open problem.

**Figure 1:**
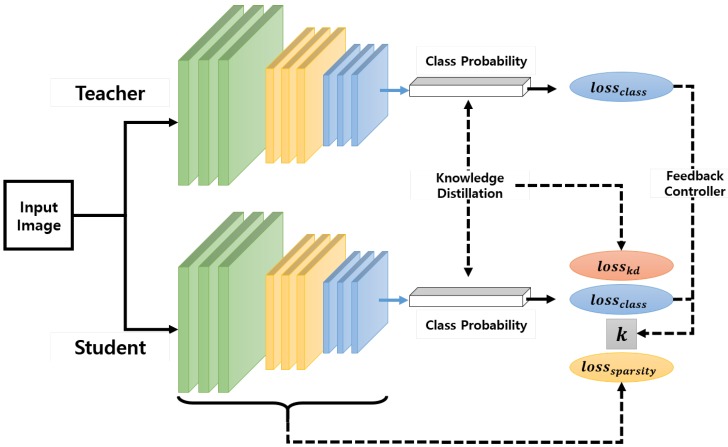
Overview.

In this paper, we propose two strategies to overcome these difficulties and design a compact network while minimizing the degradation of accuracy, as shown in Figure 1:

- The first strategy is determining the number of parameters of a student network automatically by utilizing group sparsity combined with knowledge transfer in the training process. It is already well known that a student network with a small number of parameters trained by using knowledge distillation (KD) loss leads to a better solution than those without KD. Our intuition at this point is that after the number of parameters of the student network is reduced based on a sparse loss, the resulting network can be regarded as a smaller network and the performance of such a compact network can be improved by the KD loss.
- The second strategy is to control the regularization parameter for balancing the accuracy and sparsity losses. For achieving this, we utilize a feedback control mechanism based on the proportional control theory [24,25,26,27], where the controller output is proportional to the difference between the desired setpoint and a measured value from a process (error signal). In our case, one of our goals is to make the student model mimic a cumbersome teacher model. Considering this goal in terms of control, the desired setpoint and measured process variable can be set to the accuracy losses of the teacher and student models, respectively. In addition, the feedback mechanism corresponds to adjusting the weight of the sparsity loss, i.e., the amount of emphasis put on the sparsity during the training.

Due to our assumption that the embedded sensors are low-capacity processing units, we evaluated the proposed algorithm on small-sized image datasets, i.e., CIFAR-10, CIFAR-100 [28], and ImageNet32×32 datasets [29]. The results of our experiments on these three datasets show that the proposed method helps to build a compact network by preventing performance degradation when using sparse regularization and improves the classification performance even better than KD.

This paper comprises the following sections. Section 2 describes related works on the utilization of KD, sparsity-regularized methods to obtain a compact network, and adaptive regularization. Section 3 illustrates the proposed method with KD and sparsity losses and then introduces the proposed control method for adjusting the regularization parameter to achieve a balance between the accuracy and compactness of a deep network. The detailed results of the conducted experiments are shown in Section 4. Section 5 presents the conclusions of this paper.

## 2. Related Work

It is well known that deep neural networks have many redundant parameters, and can be replaced with more compact architectures. The main problem in deploying a compact network is the increased difficulty in training the network even though the expressive power of the model is sufficient. There are two types of approaches to design a small model.

### 2.1. Knowledge Distillation

Hinton et al. [12] proposed the very first “knowledge distillation” method. In their seminal paper, they eased the training of deep networks by following a student-teacher paradigm, where the student model is trained based on the teacher network’s output, which can be viewed as a softened version of the ground-truth label, alongside the true classification label. Romero et al. [13] extended this idea by allowing the training of a deeper and thinner student than the teacher by using not just the outputs but also the intermediate representations learned by the teacher network as a hint for the student network to improve the KD. Zagoruyko et al. [14] proposed a method inspired by the attention mechanism that plays a significant role in the human visual system. In their work, the attention of a convolutional neural network (CNN) is defined as the aggregated information of the channel responses from an intermediate layer, i.e., attention maps, and a student network is forced to mimic the attention maps of a powerful teacher network. Recently, there have been efforts to use generative adversarial schemes for KD [16] and a learning strategy for one-stage online distillation [17]. But most of the studies assume that the number of parameters of the student network is fixed beforehand.

### 2.2. Parameter Reduction

This approach allows us to learn a compact structure directly from a bigger deep neural network (DNN). Alvarez et al. [19] introduced an approach to automatically determine the number of neurons in each layer of a deep network during learning. Starting from an overcomplete network, they reduced the number of parameters with a group sparsity regularizer. Wei et al. [20] also proposed a group sparsity learning method to regularize the DNN structures (i.e., filters, channels, filter shapes, and layer depth). Yoon et al. [9] proposed an exclusive sparsity regularization approach based on both $l_{1,2}$-norm and $l_{2,1}$-norm, which exploits both the positive and negative correlations among the features to enforce sparsity on the network, and removes any redundancy among the features to help utilize the full capacity of the network. However, one main problem with this approach is that it often achieves this efficiency at the expense of accuracy.

### 2.3. Adaptive Regularization

Adaptive regularization itself is a long-studied subject in various fields [30]. Several attempts have been made to apply adaptive regression for sparse learning. Zhang et al. [31], proposed a dynamic shrinking scheme for adaptive l1-regularization. Dong et al., [32] applied adaptive sparse domain selection and adaptive regularization to image blurring and super-resolution problems. There have also been attempts to apply adaptive regularization to neural networks [33,34,35]. Larsen et al. [33] proposed a method to minimize the estimate of the generalization error, which is obtained by cross-validation, by adjusting the weight decay parameter. On the other hand, Leung and Chow [34] discretely changed the parameter whenever it seems that the optimization stalls in a suboptimal solution, i.e., the gradients of the data term and the regularization term are not zero. Cho et al. [35] proposed a method to update the regularization parameter based on a diffusion process driven by a heat equation. However, all these methods focus on determining the traditional neural network parameters such as weight decay to improve generalization methods. As far as we know, there is yet an attempt to formulate adaptive regularization based on knowledge distillation for determining the sparsity of a neural network, as proposed in this paper. The proposed method is fundamentally different from the existing works in two aspects: (i) the goal of adaptive regularization in the proposed method is to determine the sparsity of neural networks, and (ii) it brings information from other (teacher) models for the control of the regularization parameter.

## 3. Proposed Method

This section details the proposed student-teacher framework with group sparsity and proportional control. First, we review the representative KD method proposed by Hinton et al. [12]. Second, we combine it with a group sparsity regularizer on the student network to find a network smaller than the initial one throughout the training phase. Finally, we show how to use the proportional control theory to balance the accuracy and sparsity losses.

### 3.1. Review of Knowledge Distillation Based on a Soft Target Distribution

The main idea of [12] was to allow the student network to learn the soft target distribution produced by the teacher network as well as the true label. The framework can be summarized as follows.

Let *T* and *S* be the teacher and student networks, respectively, and I be an indicator for representing either the teacher or the student, i.e., I∈{T,S}. Then, the class probabilities produced by a softmax output layer, a typical output of a neural network, can be represented as $p_{I}=\mathrm{softmax}\left(a_{I}\right)$, where $a_{I}$ is the vector of the pre-softmax activations of a neural network. Hinton et al. [12] introduced a relaxation parameter τ≥1 to soften the output of the neural network, i.e., $p{\left(\tau \right)}_{I}=\mathrm{softmax}\left(a_{I}/\tau \right)$, thereby allowing more information to be transferred from the teacher to the student network during training.

To force the student network to learn the pre-trained teacher’s output, as well as the true label, the weighted average of the two-loss functions is used as follows:

(1) $$L_{KD}\left(W_{S}\right)=H\left(y_{true},p_{S}\right)+\lambda_{kd}H\left(p_{T}\left(\tau \right),p_{S}\left(\tau \right)\right),$$

where $W_{S}$ denotes the collection of weights in the student network, H(·) indicates the cross-entropy, $\lambda_{kd}$ is a tunable parameter to balance the two loss terms, and $y_{true}$ is the true label of a training sample.

However, knowing the right number of parameters in advance for a student network is difficult in practice, i.e., it requires many trials and errors to arrive at the appropriate number of parameters for a student. To address this issue, we introduce the first strategy: incorporating a sparsity regularizer in the optimization. Starting from the next section, we will omit the subscript *S* in $W_{S}$ because the only weights being optimized in the proposed method are those of the student network, not those of the teacher’s.

### 3.2. Knowledge Distillation With Group Sparse Regularization

To explain the first strategy of the proposed method for designing a compact network, we first define a sequence of 4-D tensors as the weights of convolutional layers. Let *L* be the number of convolutional layers and $W^{\left(l\right)}\in R^{M_{l}\times K_{l}\times C_{l}\times N_{l}}$ be the *l*-th (1≤l≤L) weight tensor, where $M_{l},K_{l},C_{l}$, and $N_{l}$ are the dimensions along the axes of spatial height, spatial width, channel, and filter, respectively. Then, the proposed optimization objective with KD and group sparsity regularization can be formulated as:

(2) $$\arg\min_{W}L_{KD}\left(W\right)+\lambda_{r}\Sigma_{l=1}^{L}R\left(W^{\left(l\right)}\right),$$

where R(·) is the group sparsity regularization on each layer. One such candidate for the sparse regularization is the $l_{1}$-norm. However, such non-grouped sparsity regularization addresses each neuron in a layer independently, often producing inconsistent connectivity in a deep network. This can produce an irregular memory access pattern, which adversely impacts the practical acceleration performance of the hardware platform [20]. Therefore, we use the *group lasso* instead for regularization, which can be represented as

(3) $$R\left(W^{\left(l\right)}\right)=\Sigma_{g=1}^{G}{∥W_{g}^{\left(l\right)}∥}_{F}=\Sigma_{g=1}^{G}\sqrt{\Sigma_{i=1}^{|W_{g}^{\left(l\right)}|}{\left(w_{g,i}^{\left(l\right)}\right)}^{2}},$$

where ${∥A∥}_{F}$ is a Frobenius norm of A, $W_{g}^{\left(l\right)}$ is a group of partial weights in $W^{\left(l\right)}$, and *G* is the total number of groups. Minimizing (3) has the effect of reducing the number of non-zero groups, which can help in reducing the intrinsic complexity of the model and prevent random access to memory.

**Figure 2:**
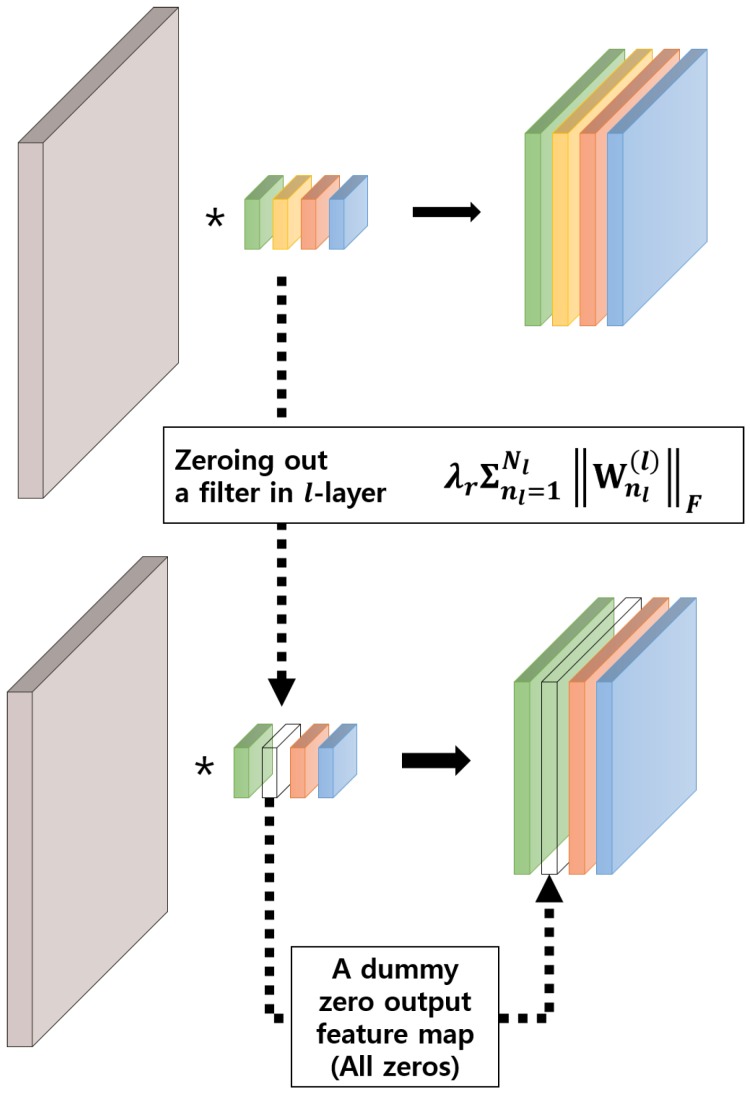
A graphical illustration of a filter-wise group sparsity regularization. Notably, zeroing out a filter in the *l*-th layer results in a dummy zero output feature map, which in turn makes a corresponding channel in the (*l* + 1)-th layer useless.

There are several options for penalizing unimportant neurons by learning the “structure”, which is decided by how the groups of $W_{g}$ are defined. In this paper, we formulate it as filter-wise group sparsity because the reduced computation is proportional to the percentage of the removed filter [20]. This has an effect of automatically deciding the number of channels used in each layer. Notably, zeroing out a filter in the *l*-th layer results in a zero-output feature map, which in turn makes a corresponding channel in the (l+1)-th layer inactive as shown in Figure 2.

Let us assume that $W_{n_{l}}^{\left(l\right)}$ is the $n_{l}$-th filter in the *l*-th layer. Then, the objective function with the filter-wise group sparsity is defined as

(4) $$\arg\min_{W}L_{KD}\left(W\right)+\lambda_{r}\Sigma_{l=1}^{L}\left(\Sigma_{n_{l}=1}^{N_{l}}{∥W_{n_{l}}^{\left(l\right)}∥}_{F}\right).$$

As can be seen in (4), this objective contains both the accuracy (including KD) and sparsity terms. It can be solved using the proximal gradient descent method [36]. This particular method is considered efficient for optimizing non-smooth functions using proximity operators [36]. In our case, the proximity operator can be represented as

(5) $$prox_{GL}\left(W^{\left(l\right)};\lambda_{r}\right)≜\arg\min_{W^{\left(l\right)}}R\left(W^{\left(l\right)}\right)+\frac{1}{2\lambda_{r}}{∥W^{\left(l\right)}-{\hat{W}}^{\left(l\right)}∥}_{F}^{2},$$

where $\lambda_{r}$ is the regularization parameter and ${\hat{W}}^{\left(l\right)}$ is the intermediate solution of $W^{\left(l\right)}$ after taking a gradient step computed only on the accuracy term, i.e., $L_{KD}\left(W\right)$, in (4). Thereafter, the proximity operator optimizes for the sparsity term by performing the Euclidean projection of the intermediate solution into the solution space. When $R(W^{\left(l\right)})$ in (4) is a group sparsity regularizer, the above problem has a closed-form solution [37]:

(6) $${W_{n_{l}}^{\left(l\right)}}^{*}={\left(1-\frac{\lambda_{r}}{∥{\hat{W}}_{n_{l}}^{\left(l\right)}{∥}_{F}}\right)}_{+}{\hat{W}}_{n_{l}}^{\left(l\right)},$$

where $n_{l}$ is the index of a group, i.e., a filter in the *l*-th layer in our case. As one can see in (6), the regularization parameter $\lambda_{r}$ plays a role in deciding how many zero groups are included in the solution. Greater the $\lambda_{r}$, the more sparse the solution. Because there is the trade-off between sparsity and accuracy, the method of selecting the value of $\lambda_{r}$ is important, but unfortunately, it is still an open question.

To overcome this difficulty, we focus on the teacher-student relationship. There is a chance that the accuracy loss from a teacher network in (4) can be used as a hint to control the regularization parameter, as explained in the next section. Intuitively, if the student model is significantly different in accuracy from the teacher, it may be more important to focus on minimizing the accuracy loss in the training process rather than enforcing the student network to be smaller. Conversely, if the student model is closer in accuracy to the teacher model, we can focus on making a smaller network. To reflect this intuition, we utilize the proportional control theory explained in the next section.

### 3.3. Proportional Control of Group Sparsity Regularization

**Figure 3:**
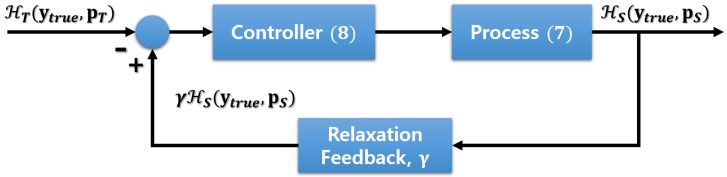
A graphical illustration of the feedback loop for adjusting the regularization parameter $\lambda_{r}$.

The objective of developing a control model based on the control theory is to find a control action that stabilizes a continuously operating dynamical system. To achieve this, a feedback control loop is designed based on the difference, i.e., error signal, between the desired value (set point, SP) and the measured value (process value, PV) [24]. Following the control theory, we set the SP and PV to the loss values obtained from the teacher and student models, respectively. Then, the loss function for training and the update equation for the control variable *k* become

(7) $$L_{KD}\left(W\right)+\exp\left(-k\right)\cdot \lambda_{r}\Sigma_{l=1}^{L}R\left(W^{\left(l\right)}\right),$$

(8) $$k\leftarrow k+\lambda_{k}\left(\gamma H_{S}\left(y_{true},p_{S}\right)-H_{T}\left(y_{true},p_{T}\right)\right),$$

respectively, where H(·) is the cross-entropy between the output of a network and the label, as explained in Section 2.1. We introduce a hyper-parameter γ∈[0,1], which is a relaxation variable to set the goal of the student slightly less than the performance of the teacher. This concept allows us to balance the effort allocated to the sparsity and the accuracy so that neither wins over the other [38]. Algorithm 1 describes the actual procedure for training the student and adjusting *k*. It is noteworthy that the algorithm is described assuming that only a single sample is used in each iteration; but in reality, each iteration is based on a mini-batch. Moreover, the adjustment of *k* is done only once in every epoch to prevent any instability in the training procedure. The values of $\left(\gamma H_{S}\left(y_{true},p_{S}\right)-H_{T}\left(y_{true},p_{T}\right)\right)$ calculated from all mini-batches are averaged for each epoch to update the variable *k*. Figure 3 shows a graphical illustration of the feedback loop for adjusting the regularization parameter.

**Algorithm 1** Optimization with a closed-loop feedback controller.
**INPUT:**W, $\lambda_{r}$, *k*, γ, learning rate ηInitialize W.**for** each epoch **do**  **for** each iteration **do**    **for** each layer *l* of a sparsity target **do**      ${\hat{W}}^{\left(l\right)}\leftarrow W^{\left(l\right)}-\eta \nabla L_{KD}\left(W^{\left(l\right)}\right)$
      $W^{\left(l\right)}\leftarrow prox_{GL}\left({\hat{W}}^{\left(l\right)};\eta \exp\left(-k\right)\cdot \lambda_{r}\right)$
    **end for**  **end for**  Update the control variable *k* using (8)
**end for**



In essence, this can be thought of as a form of closed-loop feedback control implemented using a variable *k*, thereby allowing us to control the amount of emphasis put on the sparsity cost during the gradient descent. We initialize *k* = 0. $\lambda_{k}$ is the proportional gain for *k*. In machine learning terms, it is the learning rate for *k* [38]. The regularization parameter should be changed slowly, so we use an integrated control value as shown in (8), and the controller is designed as exp(−k) to ensure that the regularization parameter is non-negative. This control, the same as our intuition described earlier, is as follows.

- At the start, *k* is set to zero, and the control value exp(−k) is set to one so that the regularization parameter is $\lambda_{g}$.
- If the performance of the student model is estimated to be worse than that of the teacher during the training, i.e., the cross-entropy value (the feedback value) from the student network is larger than that of the teacher, exp(−k) is reduced to focus more on performance enhancement.
- However, if the student’s performance is not bad compared with that of the teacher, i.e., $\gamma H_{S}\left(y_{true},p_{S}\right)-H_{T}\left(y_{true},p_{T}\right)$ is near zero, the value of exp(−k) is maintained at the same level, so that the relative strength for sparsity terms remains more or less the same. If the performance of the student is estimated to be better than that of the teacher, exp(−k) is controlled so that the overall sparsity is increased.

## 4. Experiments

### 4.1. Implementation and Environment Details

**Figure 4:**
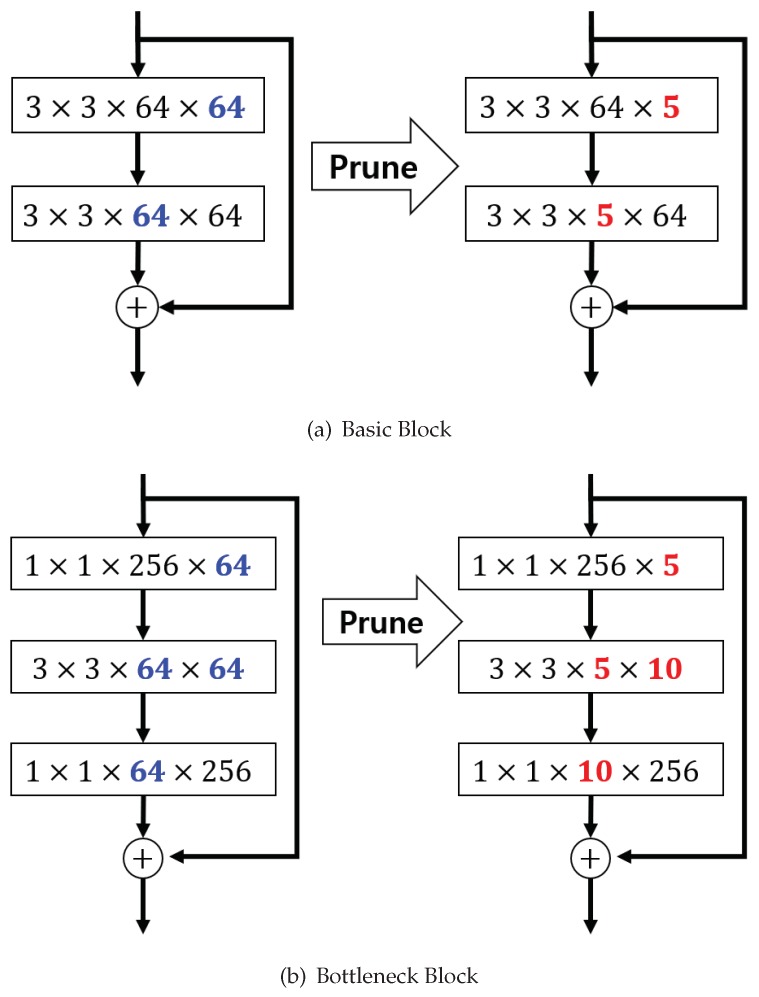
An example of a pruning ResNet. The red values are the number of remaining filters/channels.

All the experiments were performed using residual networks with three modules as used for analysis in [8], wherein the network inputs are 32 × 32 images with per-pixel mean subtraction. To measure the accuracy for a wide range of sparsity levels, we conducted the experiments by setting $\lambda_{r}$ in the following range: $0.0001\times 1.5^{i}$ where i=6,7,…,21 (Originally, the range of *i* was 0,1,⋯,21 but a small *i* did not have much significance in our experiments.), and the $\lambda_{r}$ values ranged roughly between 0.001 and 0.5. We did not apply the sparsity constraint to the last convolutional layer in each residual block as was done in [39] because the residual block has a shortcut connection as shown in Figure 4. In other words, to perform the sum operator, the last convolutional layer and the projection shortcut layer must have the same number of output feature maps. (see [8] for more details). Throughout the experiments, sparsity was measured by the number of zero-valued parameters (after training) divided by the total number of parameters. We used random crops and horizontal flips for data augmentation and normalized an input image based on the mean (0.4914, 0.4822, 0.4465) and the variance (0.2023, 0.1994, 0.2010). Following [12], the number of temperature values, i.e., τ, of the KD loss were fixed at three in all experiments. We used SGD with Nesterov momentum and set the momentum at 0.9. The learning rate η was initially set at 0.1 and was then dropped to 0.01 and to 0.001 at the half and the 3/4 points, respectively, of the entire training session. The momentum used in the study was 0.9 and the weight decay was set at 0.0001.

### 4.2. Evaluation of the Proposed Strategies

This section demonstrates the effectiveness of the proposed method through experiments. The experiments were performed on CIFAR-10, CIFAR-100 [28], and ImageNet32×32 datasets [29] using residual networks with various depths.

#### 4.2.1. CIFAR-10 Dataset

**Figure 5:**
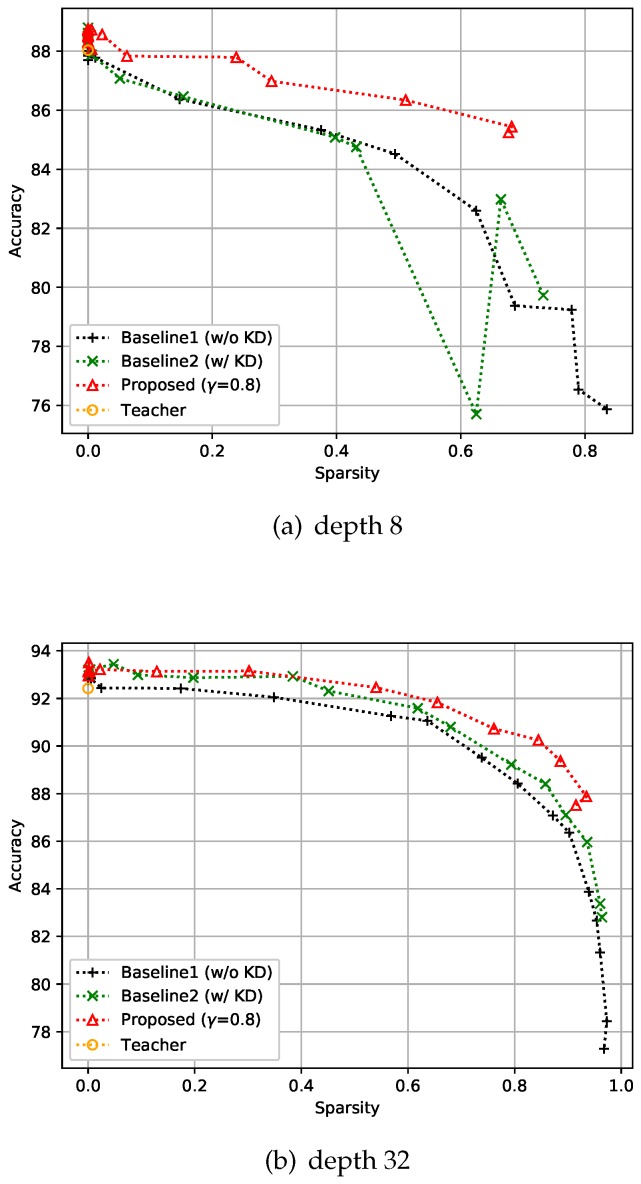
Effect of the proposed strategies on CIFAR-10. Here, the hyper-parameter γ is the relaxation variable shown in Equation (8).

CIFAR-10 is an image classification dataset with 10 classes, and comprises 50k training images and 10k test images with 32×32 resolution. For this dataset, training was performed with 128 batch size, and all the compared methods including the baseline models and the teacher model were trained over 150 epochs. The experiment was designed to verify that the proposed control-based sparsity regularization has better results in the expected trade-off of accuracy and efficiency compared with the baseline methods. The first baseline method uses only sparse regularization without KD, which is denoted as Baseline1 (w/o KD) in the figures, and the second baseline method includes both KD and sparse regularization but does not have the proposed feedback control, which is denoted as Baseline2 (w/ KD) in the figures. With these baseline settings, we conducted two experiments by changing the depth of the networks, i.e., to eight and 32, as shown in Figure 5. As expected, we can observe that the methods that include KD tend to give more accurate solutions than the sparsity-only methods. However, Figure 5a shows that the combination of KD and sparsity losses (Baseline 2) may have unstable results when there is no control. Conversely, both Figure 5a and Figure 5b show that the proposed approach involving a feedback control consistently gives more accurate results than the baselines and the results are stable even with the change of sparsity level. As reported in recent literature, when the sparsity of the network is close to zero, we can observe that KD helps to achieve better performance than the teacher.

#### 4.2.2. CIFAR-100 Dataset

**Figure 6:**
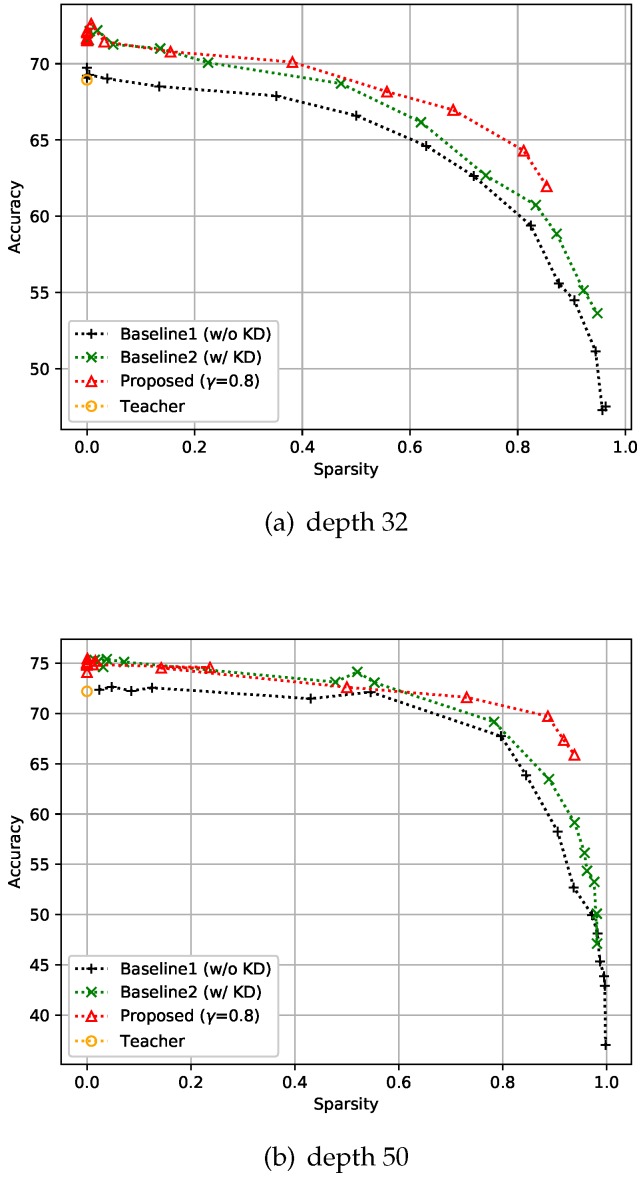
Effect of the proposed strategies on CIFAR-100. Here, the hyper-parameter γ is the relaxation variable shown in Equation (8).

We conducted experiments similar to CIFAR-10 on the CIFAR-100 dataset also. This dataset also consists of 50k training images and 10k test images with 32×32 resolution, but has 100 classes instead of 10. Our experimental batch size for training was 128, and all the compared methods, including the teacher model, were trained over 150 epochs. The depth of the networks was either 32 or 50 in this experiment. We used basic blocks for depth 32 models, and bottleneck blocks for depth 50 models, as shown in Figure 4. It is evident that the proposed method still gives better accuracy than the two baselines as shown in Figure 6. In addition, the sparser the model is, the more the improvement that can be observed under the proposed control scheme. This means that the proposed feedback control helps find a better solution by controlling *k* in the optimization process.

#### 4.2.3. ImageNet32×32 Dataset

**Figure 7:**
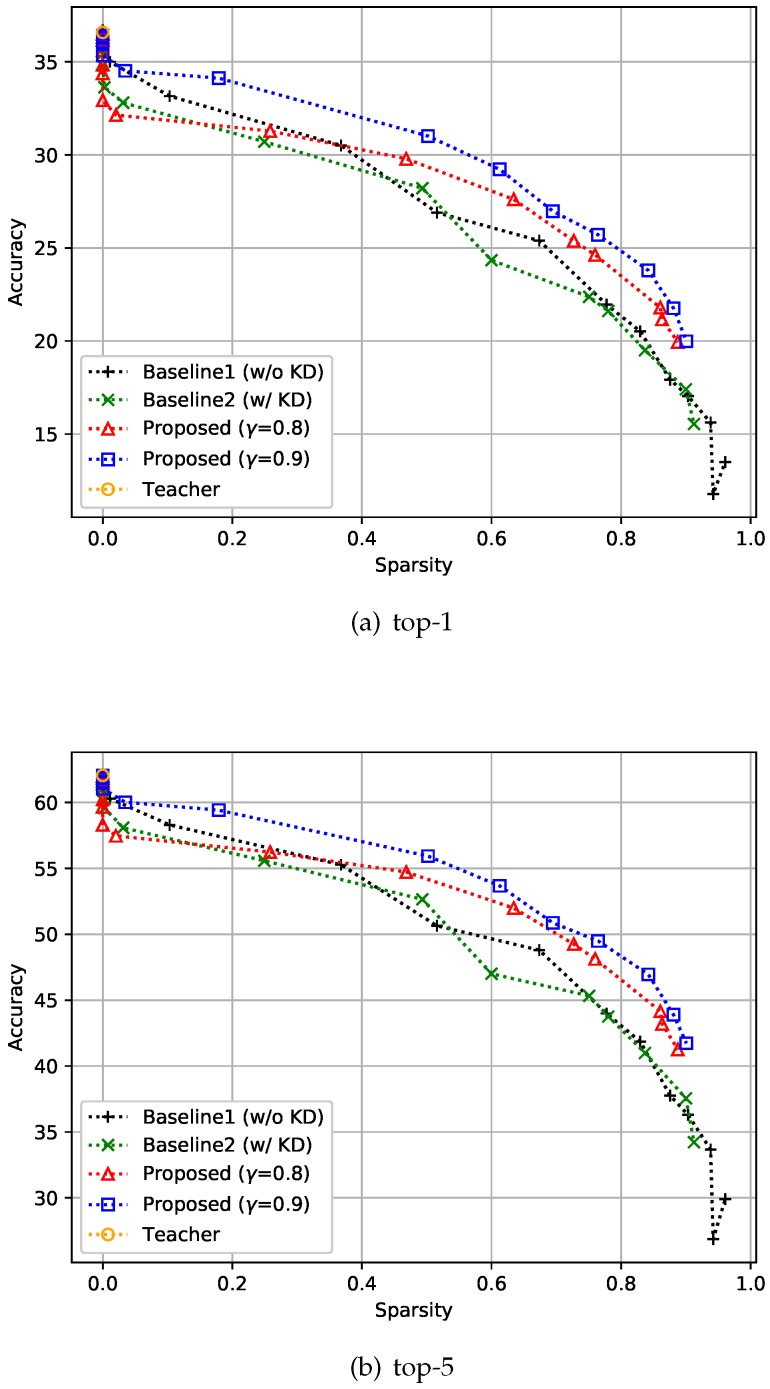
Effect of the proposed strategies on ImageNet32×32. Here, the hyper-parameter γ is the relaxation variable shown in Equation (8).

Similar to the previous datasets, experiments were conducted on ImageNet32×32 [29]. ImageNet32×32 is a 32×32-downsampled version of the ImageNet dataset [40]. This dataset is a classification dataset with 1000 classes and comprises 1,281k training images and 50k validation images. Training was performed with a batch size of 256, and all the compared methods, including the teacher model, were trained over 40 epochs. We set the depth to 32 and compared the top-1 and top-5 performances of the proposed method with the two baselines. For this experiment, KD-based methods showed slightly worse performance than non-KD-based methods when sparsity was near zero, regardless of whether feedback control was applied or not, as shown in Figure 7. Unlike in the cases of the CIFAR-10 and CIFAR-100 datasets, the teacher network on the ImageNet32×32 dataset did not achieve sufficient accuracy in the top-5, which means that the students should focus more on accuracy than on sparsity. This can be achieved by adjusting γ, i.e., increasing this variable value will set the accuracy goal of the student higher. As expected, you can see that the accuracy of the proposed method improved over that of the baselines when γ was increased.

### 4.3. Analysis of Hyper-Parameter γ

**Figure 8:**
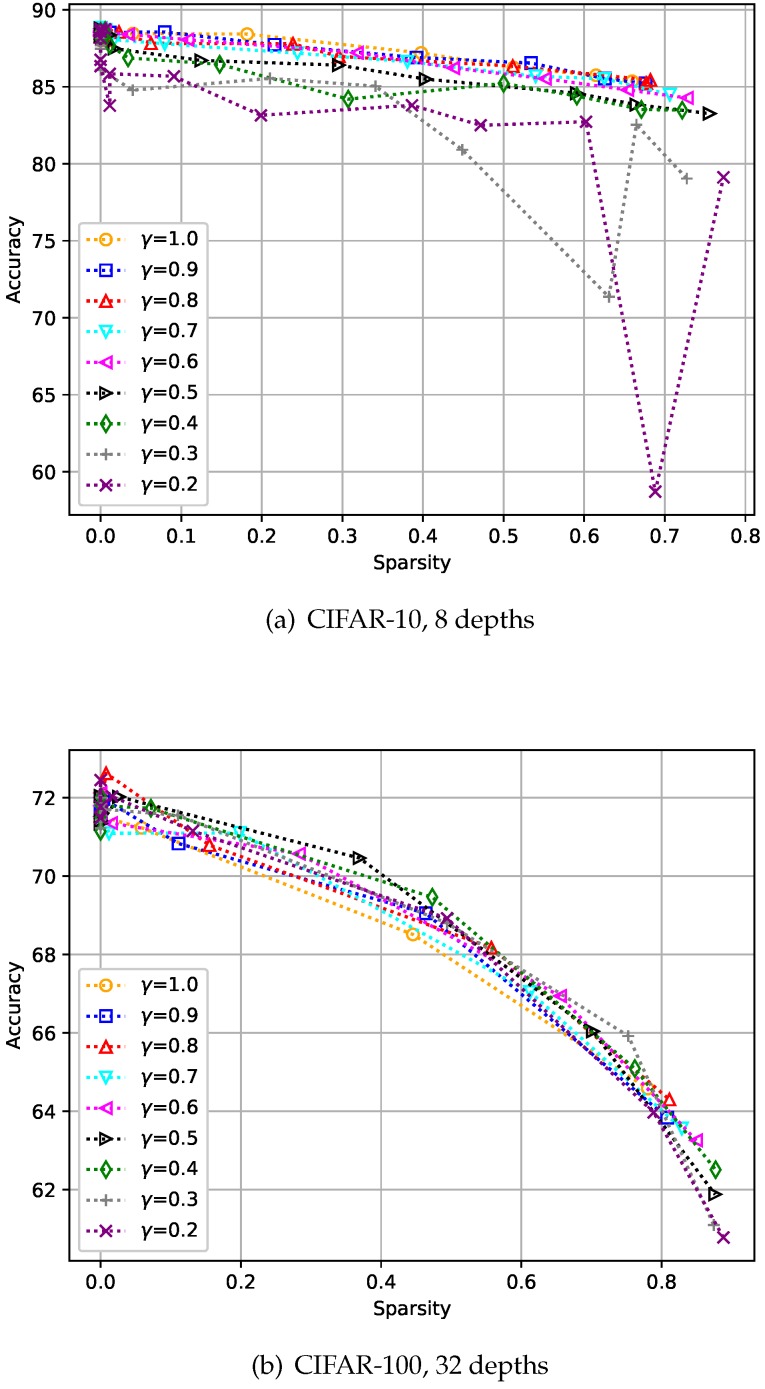
Analysis of the hyper-parameter γ. Here, the hyper-parameter γ is the relaxation variable shown in Equation (8).

We evaluated the change in performance according to the change in γ on the CIFAR-10 and CIFAR-100 datasets [28]. Here, the depths were set to eight and 32 for CIFAR-10 and CIFAR-100, respectively. Figure 8 shows the results, and we can see a γ value of 1.0 does not always give the best accuracy, as shown in Figure 8b. As intended, a smaller γ allows the student to avoid putting too much emphasis on improving its accuracy, and we chose γ of 0.8 through two experiments.

## 5. Conclusions

In this paper, we investigated the effects of two strategies for building a compact network. We demonstrated that (1) incorporating knowledge distillation when compressing the network with group sparsity achieves better performance than in other cases: a student network trained by the proposed strategies achieved better accuracy than when trained by a model with the same sparsity. (2) Moreover, it was observed that the second strategy based on proportional control can adjust the level of sparse regularization, thereby leading to significantly better results. The proposed strategies can make the application of deep networks more practical on intelligent sensors in resource-constrained platforms.
